# Comparison of the effects of histidine-triptophan-ketoglutarate solution and crystalloid cardioplegia on myocardial protection during pediatric cardiac surgery

**DOI:** 10.1186/cc13368

**Published:** 2014-03-17

**Authors:** S Kuslu, P Zeyneloglu, A Pirat, A Camkiran, M Ozkan, G Arslan

**Affiliations:** 1 Baskent University Faculty of Medicine, Ankara, Turkey

## Introduction

The major components of myocardial protection during cardiac surgery are the combination of cardioplegia solutions with hypothermia. The primary endpoint of this study is to compare the effects of histidine-triptophan-ketoglutarate (HTK) solution and crystalloid cardioplegia on release of cardiac troponin-I (cTn-I) and creatine kinase-myocardial band (CK-MB), which are perioperative determinants of myocardial protection; the secondary endpoint is to evaluate the intraoperative and postoperative hemodynamic variables and clinical outcome parameters.

## Methods

A total of 66 children aged 1 month to 6 years undergoing elective congenital heart surgery were randomly allocated to HTK solution (Group H, *n *= 32) or crystalloid cardioplegia (Group C, *n *= 34) after aortic cross-clamping. Blood samples for cTn-I and CK-MB levels were measured before the surgical incision, at the end of surgery and at 4, 16, 24 and 48 hours postoperatively.

## Results

Demographic features were similar in both groups. Duration of surgery, aortic clamp and cardiopulmonary bypass times, amounts of intraoperative fluids used and urine outputs were similar between the groups. The groups were not significantly different in terms of cTn-I and CK-MB levels at the intraoperative and postoperative period (*P *> 0.05 for all). The dose of positive inotropic drug at the end of surgery was significantly high in Group H (*P *= 0.01). The requirements for defibrillation were similar in both groups. There were no significant differences between the groups regarding postoperative hemodynamic parameters, positive inotropic requirements, amounts of fluids and blood given and pacemaker requirements (*P *> 0.05 for all). Duration of mechanical ventilation, lengths of ICU and hospital stay were similar in both groups.

## Conclusion

The present study demonstrated that there is no superiority of HTK solution and crystalloid cardioplegia to each other for myocardial protection during pediatric cardiac surgery.

